# Araçá-Boi Extract and Gallic Acid Reduce Cell Viability and Modify the Expression of Tumor Suppressor Genes and Genes Involved in Epigenetic Processes in Ovarian Cancer

**DOI:** 10.3390/plants14111671

**Published:** 2025-05-30

**Authors:** Felipe Tecchio Borsoi, Henrique Silvano Arruda, Amanda Cristina Andrade, Mônica Pezenatto dos Santos, Isabelle Nogueira da Silva, Leonardo Augusto Marson, Ana Sofia Martelli Chaib Saliba, Severino Matias de Alencar, Murilo Vieira Geraldo, Iramaia Angélica Neri Numa, Glaucia Maria Pastore

**Affiliations:** 1 Department of Food Science and Nutrition (DECAN), School of Food Engineering (FEA), University of Campinas (UNICAMP), Monteiro Lobato Street 80, Campinas 13083-862, São Paulo, Brazil; felipe.tecchio@gmail.com (F.T.B.); andradenut@gmail.com (A.C.A.); iramaia@unicamp.br (I.A.N.N.); glaupast@unicamp.br (G.M.P.); 2 Department of Structural and Functional Biology, Institute of Biology, University of Campinas (UNICAMP), Campinas 13083-863, São Paulo, Brazil; monica.pezenatto@gmail.com (M.P.d.S.); isabellendsa@gmail.com (I.N.d.S.); leonardoaugustomarson@gmail.com (L.A.M.); murilovg@unicamp.br (M.V.G.); 3 Department of Agri-Food Industry, Food and Nutrition, “Luiz de Queiroz” College of Agriculture (ESALQ), University of São Paulo (USP), Piracicaba 13418-900, São Paulo, Brazil; sofiasaliba@usp.br (A.S.M.C.S.); smalencar@usp.br (S.M.d.A.)

**Keywords:** *Eugenia stipitata*, phenolic extract, gene expression, *BRCA1*, DNA methylation

## Abstract

In the present study, we characterized and investigated the effect of the araçá-boi extract on antioxidant activity, cell viability, and the regulation of genes related to tumor suppression and epigenetic mechanisms in ovarian cancer cells. The results showed that araçá-boi extract revealed a remarkable diversity of phytochemicals (organic acids, phenolic acids, and flavonoids), significant antioxidant potential, and efficient scavenging of reactive oxygen species, particularly hydroxyl and peroxyl radicals. Gallic acid, one of the phenolic acids present in the extract, was used alone to verify its contribution to cytotoxic activities. Exposure of human ovarian cancer cells (NCI/ADR-RES and OVCAR3) to the extract (0.15–150 μg/mL) and gallic acid (6–48 μg/mL) resulted in a significant reduction in cell viability, particularly after 48 h of treatment. Both treatments modulated genes involved in DNA repair, tumor suppression, and epigenetic regulation. However, no changes were observed in the methylation status of the *BRCA1* gene promoter region with either araçá-boi extract or gallic acid. These findings reinforce the therapeutic potential of araçá-boi extract and its phenolic compounds against ovarian cancer and point to the need for further studies to better elucidate the molecular pathways involved and validate these effects in vivo.

## 1. Introduction

Ovarian cancer is a malignant neoplasm that originates in ovarian tissues and is known for being one of the deadliest types of gynecological cancer. It can be classified into two groups: epithelial carcinoma, which is the most common and accounts for about 90% of cases, and non-epithelial origin (germ cells and stromal cells) [1]. According to GLOBOCAN [2], ovarian cancer ranks eighth among the most common cancer types in women, with high prevalence and mortality, responsible for over 374,000 new cases and around 240,000 deaths annually. The high mortality rate associated with ovarian cancer is largely due to late-stage diagnosis, as the absence of specific symptoms in early stages and the lack of effective early detection strategies remain significant challenges, highlighting the urgency to adopt new therapeutic strategies focused on tumorigenesis and chemotherapy resistance [3].

Recent advances in omics sciences and big data, such as genomics, epigenomics, transcriptomics, proteomics, metabolomics, and machine learning, have provided new insights into the molecular mechanisms of ovarian cancer. These tools are essential for identifying genes, proteins, and metabolites involved in the development and progression of cancer, directly impacting diagnosis, prognosis, and therapeutic options [4]. In ovarian cancer, epigenomics plays a crucial role, as alterations like DNA hypermethylation can silence tumor suppressor genes (e.g., *RASSF1A*, *CDKN2A*, *BRCA1*, *MLH1*, and *CDH1*), which not only compromises the regulation of cell growth and vital processes such as apoptosis, DNA repair, and the cell cycle, but also disrupts cell adhesion, facilitating metastasis [5,6]. Additionally, genes involved in epigenetic processes such as *DNMT1* and *HDAC1* play a central role in this context: DNMT1 adds methyl groups to DNA, perpetuating hypermethylation, while HDAC1 compacts chromatin, inhibiting the transcription of tumor suppressor genes [7]. Therefore, the use of therapies based on the inhibition of these enzymes is crucial to reverse the silencing of these genes, restoring their expression and potentially reversing tumor processes. Currently, epigenetic agents, “epidrugs”, have improved cancer treatment. However, there are still many adverse effects and challenges that limit the effectiveness of these treatments [8]. In this context, there is a growing interest in natural compounds that not only inhibit these epigenetic enzymes but also provide a less toxic alternative, promoting a cellular environment more conducive to apoptosis and cellular repair [9]. Natural compounds, such as polyphenols, mainly found in plant species, demonstrate great potential in the treatment of various types of cancer. These compounds are capable of affecting different cancer-related mechanisms, such as cell proliferation, inflammation, invasion, and metastasis [10,11]. Along with all these benefits, their ability to affect epigenetic processes is one of the most important aspects of their impact. Recent studies indicate that polyphenols act as epigenetic modulators by interfering with DNA and histone methylation and acetylation pathways. These alterations promote the reactivation of tumor suppressor genes, helping to control cancer progression [9,12]. Thus, polyphenols have the potential not only to prevent but also to reverse harmful epigenetic changes, reinforcing their role as promising therapeutic agents in oncology.

*Eugenia stipitata* Mac Vaugh, popularly known as araçá-boi, is a fruit native to the Amazon, cultivated in countries such as Brazil, Peru, Bolivia, Ecuador, and Colombia [13]. The araçá-boi fruit (peel and pulp) is rich in phenolic compounds (e.g., trans-cinnamic acid, ellagic acid, gallic acid, syringic acid, myricetin, quercetin, and kaempferol derivatives), carotenoids, and vitamins [13,14,15,16]. Previous studies have already indicated that the phenolic compounds present in the araçá-boi fruit have antioxidant, anti-inflammatory, cytotoxic, and anticancer potential effects [15,16,17]. Among the phenolic compounds present in araçá-boi, gallic acid is one of the major compounds and has stood out due to its effects on cytotoxic activity in ovarian tumor cells [18,19,20]. Considering these aspects, this compound was selected as a comparative control to the araçá-boi extract in order to infer whether the extract’s potential activity could be related to the presence of this compound. Additionally, the specific effects of araçá-boi extract and gallic acid on ovarian cancer cells, particularly regarding the modulation of tumor suppressor genes and epigenetic mechanisms, have not been thoroughly investigated. In light of this, the present study seeks to characterize the phytochemical profile of the araçá-boi extract and investigate its effects on antioxidant activity, cell viability, gene modulation, and DNA methylation in ovarian cancer cells.

## 2. Results and Discussion

### 2.1. Total Phenolic Content (TPC), Total Flavonoid Content (TFC), and Antioxidant Capacity of Araçá-Boi Extract

The results for the total phenolic compounds (TPC), total flavonoid content (TFC), and antioxidant capacity of the araçá-boi extract are shown in Table 1. The TPC and TFC from araçá-boi extract were 25.90 mg GAE/g dw and 6.53 mg CE/g dw, respectively. According to Rufino et al. [21], food matrices are categorized by dry weight (dw) into low (<10 mg GAE/g), medium (10–50 mg GAE/g), and high (>50 mg GAE/g) levels of TPC. Therefore, the araçá-boi extract obtained in this study can be considered a good source of phenolic compounds since it exhibits a medium content of these phytochemicals. These results surpass those reported by de Araújo et al. [14] and Llerena et al. [22], who found for the edible fraction of araçá-boi TPC values of 9.06 and 15.65 GAE/g dw and TFC values of 1.25 and 6.00 mg CE/g dw, respectively. Variations in TPC and TFC among studies can be attributed to a combination of methodological, environmental, and agronomic factors, including storage conditions, sample preparation, extraction methods, edaphoclimatic conditions, cultivation practices, and harvest timing, which collectively influence the stability, recovery, and phytochemical composition of plants [23,24].

Antioxidant activity is positive and strongly linked to the phenolic compounds present in the plant matrix [25]. These compounds have many phenolic hydroxyl groups (Phenyl-OH or for aromatics, Aryl-OH) which are planar and electron-rich, being able to act by reducing or inhibiting reactive species through hydrogen atom transfer and/or single electron transfer. These abilities of phenolic compounds can prevent or reduce oxidative stress-related diseases, cardiovascular diseases, chronic respiratory diseases, diabetes, cancers, and mental illness [26]. Thus, the antioxidant capacity of araçá-boi extract was evaluated both for the scavenging of synthetic free radicals and reactive oxygen species. As shown in Table 1, the araçá-boi extract demonstrated remarkable peroxyl radical (ROO^•^) scavenging capacity, with a value of 366.13 µmol TE/g dw. This capacity was followed by similar values obtained in the FRAP and ABTS assays, recording 161.77 and 155.52 µmol TE/g dw, respectively. In contrast, the antioxidant activity measured by the DPPH assay was lower, reaching only 66.78 µmol TE/g dw. A recent study by Borsoi et al. [16] found a similar trend in araçá-boi extract, with a higher value for peroxyl radical scavenging (583.81 µmol TE/g dw) and lower values for the ABTS and FRAP methods (102.51 and 150.77 µmol TE/g dw). On the other hand, Soares et al. [17] reported a value of only 32.72 µmol TE/g dw for peroxyl radical scavenging in araçá-boi pulp. Similarly, de Araújo et al. [27] found values of 8.40, 25.30, and 22.80 µmol TE/g dw for the DPPH, ABTS, and ROO^•^ methods, respectively. Antioxidant methods are based on different mechanisms of action, including single electron transfer (e.g., DPPH and FRAP), hydrogen atom transfer (e.g., ROO^•^), or mixed-mode assays (e.g., ABTS) [28,29]. This indicates that the phenolic compounds in the araçá-boi extract exert antioxidant activity more efficiently through the hydrogen atom transfer mechanism.

In addition to ROO^•^, other ROS, such as hydroxyl radical (OH^•^), superoxide radical (O_2_^•−^), and hypochlorous acid (HOCl), were also investigated in this study. Considering that the IC_50_ (inhibitory concentration) represents the concentration of the extract required to reduce the oxidative effect of reactive species by 50%, the results from Table 1 show the highest elimination activity for OH^•^, followed by HOCl and O_2_^•−^ (1.91, 307.66, and 3534.33 µg/mL dw, respectively). ROS are highly reactive molecules playing an important role in biological processes such as the maintenance of cellular redox homeostasis, signal transduction, and defense against pathogens. However, when ROS production exceeds the capacity of the organism’s antioxidant system, oxidative stress occurs, resulting in damage to essential biomolecules such as DNA, RNA, proteins, and cellular membranes. This imbalance in the body can lead to the development of various chronic diseases, such as cardiovascular diseases, diabetes, neurodegenerative diseases, cancer, and inflammatory disorders [30,31,32]. It can be inferred that the araçá-boi extract has a relevant and significant antioxidant effect, being particularly effective in neutralizing OH^•^. This radical is one of the most reactive and damaging to cells, as it can interact with all biological molecules, causing cellular damage to lipids, proteins, and membranes, and there are no existing enzymatic systems to scavenge OH^•^, demonstrating the importance of obtaining sequestering agents from these species through diet [33]. However, the araçá-boi extract showed limited activity in scavenging HOCl and O_2_^•−^ due to the high IC_50_ values observed for these reactive oxygen species (307.66 and 3534.33 µg/mL dw, respectively). Soares et al. [17] evaluated different ROS and RNS in the araçá-boi pulp. The authors found an IC_50_ of 758.13, 14.64, and 6.95 µg/mL dw for O_2_^•−^, HOCl, and nitric oxide (NO^•^), respectively. HOCl generated in excess during the inflammatory response can cause tissue damage, while the O_2_^•−^ is a precursor to other free radicals and can contribute to oxidative stress when present at elevated concentrations [32,33]. Thus, the ability of the araçá-boi extract to eliminate different ROS, particularly acting as a strong scavenger of OH^•^ and ROO^•^, suggests therapeutic potential, especially in conditions associated with oxidative stress, where the increase in these radicals can compromise cellular and molecular integrity. Furthermore, these results highlight the importance of araçá-boi as a promising antioxidant modulator, with the potential to be used in the development of therapies or products that combat the harmful effects of oxidative stress.

### 2.2. Phytochemical Profile by UHPLC-Q-Orbitrap-MS/MS of Araçá-Boi Extract

The literature has shown that the edible part of araçá-boi contains a considerable number of phytochemicals with antioxidant potential against both synthetic free radicals and reactive oxygen and nitrogen species. Several phytochemicals, particularly phenolic compounds, have been associated with the antioxidant effects of araçá-boi [13,14,17]. Despite recent efforts to identify the phytochemicals in araçá-boi, there is still a limited number of studies with this focus, and a more detailed characterization has not yet been achieved. Therefore, in this work, we conducted a characterization of the phytochemical profile of the araçá-boi extract using UHPLC-Q-Orbitrap-MS/MS. For this, non-targeted metabolite profile and data processing were carried out using Xcalibur 4.3 software. The characterization strategy was based on exact mass (mass accuracy limit of 8 ppm), fragmentation patterns, and comparison with data available in the literature and existing phytochemical databases (e.g., MassBank (http://massbank.jp (accessed on 10 January 2025)), METLIN Metabolite (https://metlin.scripps.edu (accessed on 10 January 2025)), and HMDB (https://hmdb.ca (accessed on 10 January 2025))). Table 2 shows the fragmentation patterns (main MS/MS fragment ions) along with the exact masses of precursor ions in negative ionization mode, molecular formula, error (ppm), retention time (min), and tentative identification for each phytochemical found in the araçá-boi extract.

Seventy-three phytochemical compounds were tentatively annotated and characterized based on their MS and MS/MS data in the araçá-boi extract, including ten organic acids, thirty-six phenolic acids, and twenty-seven flavonoids. As observed, the araçá-boi extract possesses a wide diversity of phenolic acids (e.g., gallic, vanillic, caffeic, coumaric, ellagic acids, and/or their derivatives) and flavonoids (mainly as glycosylated forms of myricetin, quercetin, and kaempferol). In addition, it contains a considerable variety of organic acids, such as quinic, malic, citric, shikimic, succinic, ascorbic acids, etc. The presence of organic acids in the araçá-boi extract is important, not only for their contribution to the characteristic taste and acidity of the fruit, but also for their significant biological roles. Organic acids like malic acid are crucial intermediates in the Krebs cycle, essential for cellular energy production [34]. Furthermore, ascorbic acid (vitamin C) is known for its potent antioxidant properties, protecting cells against oxidative stress and contributing to collagen synthesis and immune function [35]. Other organic acids not only play an important role in nutrient absorption, but also contribute to the aroma, taste, and health benefits. These organic acids help improve the bioavailability of other bioactive compounds, such as phenolic acids and flavonoids, present in plant [36]. Phenolic compounds play a key role in plant defense against biotic and abiotic stress, but they can also be toxic to the plant. To mitigate this, plants conjugate these toxic compounds with organic molecules, such as carbohydrates, through glycosyltransferase enzymes, forming less toxic or non-toxic glycosylated forms. These compounds are stored in vacuoles until needed for defense, when they are activated by glycosylhydrolase enzymes. Glycosylation also enhances their solubility, stability, and metabolism, allowing for better distribution and accumulation in plant cells [37]. This may explain why we identified mainly glycosylated phenolic compounds in the araçá-boi extract. Similarly to our work, de Araújo et al. [14] identified a total of 18 compounds in the edible fraction of araçá-boi (pulp and peel) by ESI-LTQ-XL-MS/MS in both positive and negative modes, including only one organic acid (malic acid), phenolic acids (gallic, cinnamic, vanillic, caffeic, and coumaric acids derivatives), and flavonoids (mainly glycosylated forms of myricetin, luteolin, kaempferol, and quercetin). On the other hand, Soares et al. [17] observed a profile mainly composed of hydrolysable tannins (ellagitannins and their glycosylated derivatives), phenolic acids (ellagic acid, coumaric acid, vanillic acid, and their derivatives), and flavonoids (eriodyctiol, pinoresinol, epicatechin, quercetin, and their derivatives) through LC-ESI-QTOF-MS analysis. The phenolic acids, including gallic, cinnamic, coumaric, and ellagic acids, and flavonoids (e.g., myricetin, quercetin, and kaempferol), are renowned for their potent activity against oxidative stress and inflammation [38]. Moreover, phenolic acids and flavonoids can influence the expression of proteins and epigenetic pathways involved in cell cycle regulation, apoptosis, and DNA repair mechanisms. These actions are particularly relevant in the prevention and management of chronic diseases, such as cardiovascular diseases, diabetes, and cancer [38,39]. Therefore, the araçá-boi extract contains a wide and diverse array of phenolic compounds, which can contribute to numerous beneficial effects on human health and overall well-being.

### 2.3. Cell Viability in Healthy Chinese Hamster Ovary Cells and Human Ovarian Tumor Cells

**Figure 1:**
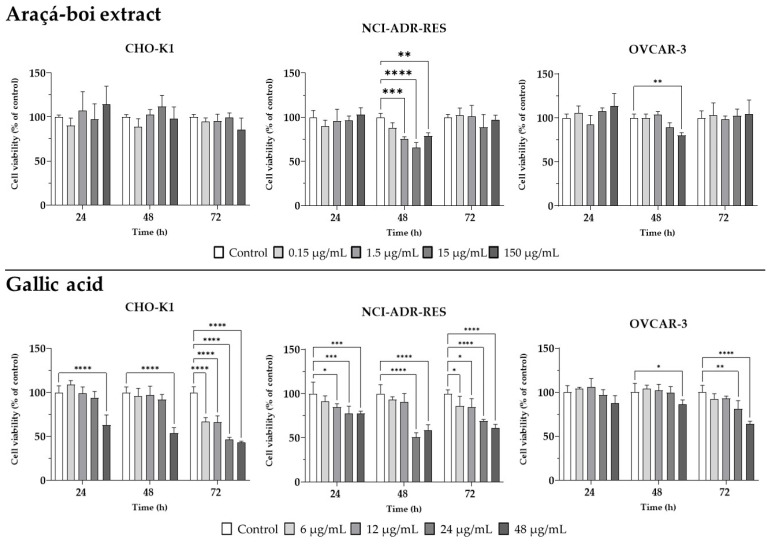
The effect of different concentrations of araçá-boi extract and gallic acid on the viability of the normal Chinese hamster ovary (CHO-K1) cell line and the human ovarian cancer cell line (NCI-ADR-RES and OVCAR3). Viability was measured by the MTT assay after 24, 48, and 72 h. Values with *p* < 0.05 were considered statistically significant. * *p* < 0.05, ** *p* < 0.01, *** *p* < 0.001, **** *p* < 0.0001.

To evaluate cytotoxicity, an MTT cell viability assay was conducted on Chinese hamster ovary cells (CHO-K1) and two human ovarian tumor cell lines (NCI/ADR-RES and OVCAR-3). The araçá-boi extract was tested at four different concentrations (0.15, 1.5, 15, and 150 µg/mL) and over three distinct exposure times (24, 48, and 72 h). In parallel, gallic acid, one of the phenolic acids present in the extract, was also tested at concentrations of 6, 12, 24, and 48 µg/mL to assess its isolated effect (Figure 1).

The araçá-boi extract did not exhibit cytotoxicity in CHO-K1 cells at any of the tested concentrations and exposure times, suggesting a safety profile for normal cells. In contrast, isolated gallic acid showed a distinct behavior, with a significant cytotoxic effect observed at the highest concentration (48 µg/mL) after 24 and 48 h of exposure. This effect became evident at all tested concentrations after 72 h, indicating a time- and concentration-dependent action for gallic acid that differs from the overall effect of the extract. For the tumor cell lines, the araçá-boi extract did not significantly reduce cell viability at 24 or 72 h for either line. However, after 48 h of exposure, a significant reduction in viability was observed for the NCI/ADR-RES line at concentrations of 1.5, 15, and 150 µg/mL, while the OVCAR-3 line showed reduced viability only at the highest concentration (150 µg/mL). In the case of gallic acid, NCI/ADR-RES cells exhibited a significant reduction in cell viability at concentrations of 12, 24, and 48 µg/mL at 24 h, 24 and 48 µg/mL at 48 h, and at all concentrations by 72 h of exposure, indicating a more immediate and potent cell viability reduction effect. On the other hand, for the OVCAR-3 line, a similar behavior to the extract was found, with a significant effect only at the highest concentration (48 µg/mL) after 48 h, and at 24 and 48 µg/mL after 72 h. These results suggest that gallic acid may partially account for the extract’s cytotoxic activity, potentially through a synergistic or antagonistic effect that modulates this reduction in cell viability. The araçá-boi extract is a complex mixture of various phytochemicals, primarily including phenolic acids and glycosylated flavonoids, as noted above. These compounds are known for their cytotoxic and anticancer effects. For instance, Neri-Numa et al. [15] observed that araçá-boi pulp extract is not cytotoxic to green monkey kidney cells. Furthermore, the same authors did not find antiproliferative activity from the extract in nine tumor cell lines, including human ovarian tumor cell lines (NCI/ADR-RES and OVCAR-3). In contrast, Borsoi et al. [16] demonstrated that araçá-boi extract and trans-cinnamic acid reduce cell viability in melanoma tumor cells (SK-MEL-28). Similar to our findings, Varela-Rodríguez et al. [20] reported that gallic acid and myricetin exhibited low selectivity, showing cytotoxic activity in both a cell line derived from normal human bronchial epithelial cells (BEAS-2B) and human ovarian tumor cells (OVCAR-3 and SKOV-3), possibly linked to the cellular phenotype. Several studies have demonstrated that phenolic plant extracts can inhibit cell viability through different mechanisms, such as inducing apoptosis, cell cycle arrest, and oxidative stress, modulating signaling pathways, altering gene expression, changing cell membranes, and promoting mitochondrial disruptions and DNA damage [40,41,42]. For instance, Homayoun et al. [43] showed that the treatment of OVCAR-3 ovarian cancer cells with grape seed extract led to a reduction in cell growth and proliferation and induction of the apoptosis process.

The absence of cytotoxic effects after 24 h of treatment with the araçá-boi extract may be influenced by several factors, including insufficient exposure time for the phenolic compounds to exert measurable effects, as well as the complexity of the extract matrix, which may result in slower cellular uptake or delayed biological activity [44]. Moreover, interactions among multiple phytochemicals in the extract may contribute to synergistic, additive, or even antagonistic effects, which can influence the overall bioactivity profile [45]. On the other hand, the action of isolated gallic acid can be explained by its nature as a single compound, which acts directly on tumor cells without interference from other components present in the extract. Gallic acid, due to its structure and bioactivity, may induce cellular damage more rapidly compared to the crude extract, which relies on synergistic or antagonistic interactions among its compounds [46]. For instance, Balushi et al. [47] observed that gallic acid decreased cell viability in a concentration-dependent manner in cisplatin-sensitive (A2780S) and resistant (A2780CP) ovarian cancer cell lines. For OVCAR-3, the more limited response observed for both the araçá-boi extract and gallic acid may be associated with more robust resistance mechanisms against these treatments, which require higher concentrations of the extract or gallic acid to be overcome [48].

Therefore, the results revealed the differential impact of araçá-boi extract and gallic acid on ovarian tumor cells (NCI/ADR-RES), particularly at 48 h of exposure, highlighting the need for further investigation into the molecular mechanisms involved. Additional analyses were performed to assess the expression of genes essential for cellular damage repair, cell cycle regulation, and epigenetic modulation. These genes play fundamental roles in tumor suppression and cellular response to therapies, providing insights into resistance mechanisms and potential targets for therapeutic interventions.

### 2.4. Relative Gene Expression of Tumor Suppressor Genes and Epigenetic Enzymes

**Figure 2:**
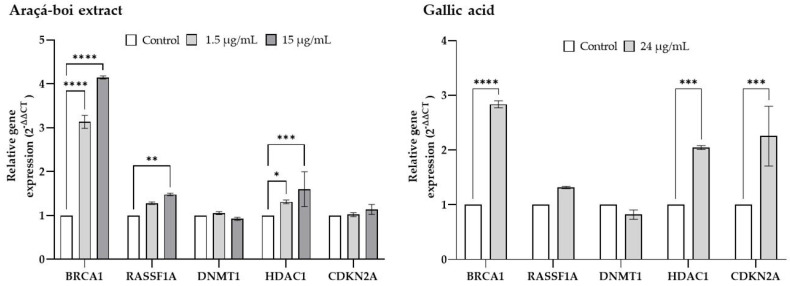
Relative gene expression 2^(−ΔΔCT)^ of NCI-ADR-RES cells treated with araçá-boi extract (1.5 and 15 μ/mL) and gallic acid (24 μg/mL) for 48 h. The relative expressions of *BRCA1*, *RASSF1A*, *DNMT1*, *HDAC1*, and *CDKN2A* were measured by RT-PCR. GAPDH was used as the housekeeping gene to normalize gene expression. Values with *p* < 0.05 were considered statistically significant. * *p* < 0.05, ** *p* < 0.01, *** *p* < 0.001, **** *p* < 0.0001.

RT-PCR was employed to assess the effects of araçá-boi extract and gallic acid on the expression levels of genes associated with DNA repair (*BRCA1*), tumor suppression (*RASSF1A*), cell cycle regulation (*CDKN2A*), DNA methylation (*DNMT1*), and histone deacetylation (*HDAC1*) on ovarian tumor cells (NCI/ADR-RES) after 48 h of exposure (Figure 2).

The results showed that treatment with araçá-boi extract significantly increased the expression of tumor suppressor genes (*BRCA1* and *RASSF1A*) and genes involved in the epigenetic process (*HDAC1*), especially at 15 µg/mL. Gallic acid (24 µg/mL), an extract component, had a more pronounced effect on *BRCA1*, *HDAC1*, and *CDKN2A* gene expression. The literature features numerous studies on isolated phenolic compounds or plant extracts with the potential to modulate tumor suppressor genes and epigenetic enzymes in cancer [6,49,50,51]. For instance, Homayoun et al. [43] observed that grape seed extract exerts cytotoxic effects in chemoresistant human ovarian cancer cells (OVCAR-3), potentially mediated by the modulation of genes involved in signaling pathways (*PTEN*, *AKT*, *mTOR*, *DACT1*, *GSK3B*, and *C-MYC*), cell cycle regulation (*CDK4* and *CCND1*), and apoptosis (*BAX*, *BCL2*, *CASP3*, *CASP8*, and *CASP9*). Another study proposed by Nowrasteh et al. [52] observed that a commercial fruit extract rich in polyphenols and flavonoids alters the expression of genes involved in epigenetic processes (*HDAC1*, *HDAC2*, *DNMT1*, *DNMT3A*, and *DNMT3B*) in a DMBA (7,12-dimethylbenz(a)anthracene) carcinogen-induced animal model and, consequently may delay cancer development and tumor progression. The upregulation of tumor suppressor genes, including *BRCA1*, *RASSF1*, and *CDKN2A*, combined with the downregulation of genes involved in epigenetic processes, such as *DNMT1* and *HDAC1*, has the potential to delay cancer onset and hinder tumor progression [53]. *BRCA1* primarily functions in maintaining genomic integrity by repairing double-strand DNA breaks, which is essential for cellular stability [54]. The *RASSF1A* gene is a tumor suppressor that regulates cell cycle, apoptosis, cell migration, cell adhesion, and microtubule stabilization [55]. Thus, the upregulation of *BRCA1* and *RASSF1* by araçá-boi extract suggests a potential activation of DNA repair pathways and an inhibitory function in signaling pathways such as Ras/MAPK, which contributes to cell cycle arrest and the promotion of apoptosis, potentially reducing the cell proliferation typical of cancer. On the other hand, gallic acid has been shown to upregulate the *BRCA1* and *CDKN2A* genes. *CDKN2A* is a tumor suppressor gene that encodes proteins such as p16, which inhibit cyclin-dependent kinase activity, thereby halting cell cycle progression, particularly at the G1 to S phase transition [56]. The upregulation of *BRCA1* and *CDKN2A* may lead to DNA repair and cell cycle arrest, contributing to a reduction in cell proliferation. Therefore, this suggests that the phytochemicals present in araçá-boi, including gallic acid, may contribute to activating this repair pathway, enhancing cellular defense against mutations that promote tumor development. Additionally, isolated gallic acid influences different molecular pathways compared to araçá-boi extract, highlighting the synergistic and/or antagonistic interactions of the phenolic compounds in the extract.

Regarding epigenetic enzymes, no changes in *DNMT1* modulation were observed, while the upregulation of *HDAC1* was recorded for both the araçá-boi extract and gallic acid. *DNMT1* is involved in maintaining DNA methylation, a process that can silence tumor suppressor genes, promoting the proliferation of cancer cells. On the other hand, HDACs, such as *HDAC1*, play a role in removing acetyl groups from histones, resulting in DNA compaction and gene transcription repression. In ovarian cancer, *DNMT1* and *HDAC1* can facilitate tumor cells’ survival by silencing tumor suppressor genes and promoting resistance to apoptosis [57]. Although no studies to date have specifically reported the effects of gallic acid on *DNMT1* suppression and *BRCA1*/*CDKN2A* upregulation in ovarian cancer cells, evidence from other tumor models suggests its promising epigenetic activity. For instance, in lung cancer cells (H1299), gallic acid significantly decreased nuclear and cytoplasmic levels of *DNMT1* and *DNMT3B*, contributing to global DNA demethylation and the reactivation of tumor suppressor pathways [58]. As mentioned above, phenolic compounds derived from plants can downregulate these enzymes, thereby restoring the expression of tumor suppressor genes. Nevertheless, the lack of *DNMT1* modulation observed in our study suggests that, while araçá-boi extract and gallic acid do not inhibit *DNMT1* activity, they also do not promote pro-tumoral epigenetic processes related to DNA methylation. On the other hand, the upregulation of *HDAC1*, although potentially associated with transcriptional repression, may disrupt pro-tumoral pathways or enhance the sensitivity of cells to *HDAC1* inhibitors. This analysis provides a comprehensive perspective on how araçá-boi extract and gallic acid may modulate critical signaling pathways in ovarian tumor cells, identifying promising molecular targets for therapeutic development. The results suggest that the phenolic compounds present in the extract not only have the potential to act specifically on targets related to cell viability and epigenetic regulation but could also be explored in combination therapies. These findings open new avenues to expand the therapeutic spectrum for ovarian cancer treatments, enhancing the effectiveness of current therapeutic approaches.

### 2.5. DNA Methylation Profiling of BRCA1 Promoter

Genomic DNA was isolated from NCI/ADR-RES ovarian tumor cells and treated with sodium bisulfite using the EZ DNA Methylation-Direct™ Kit (Zymo Research Corporation, Irvine, CA, USA) to convert all unmethylated cytosines into uracils, leaving methylated cytosines unchanged. In subsequent PCR reactions, unmethylated cytosines are read as Ts (or complementary strand As), while methylated cytosines are read as Cs (or complementary strand Gs). The modified DNA was then used as a template for PCR reactions with primers designed to amplify specific regions in the promoters of the target gene. The PCR products were purified using PCR purification columns and sequenced.

**Figure 3:**
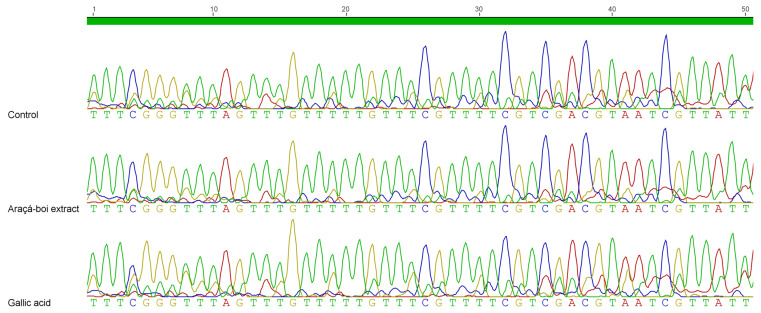
Electropherogram of the methylation status of the *BRCA1* gene promoter region in ovarian tumor cells NCI/ADR-RES treated with araçá-boi extract (15 μg/mL) or gallic acid (24 μg/mL) for 48 h. Cytosine-C, blue; Thymine-T, green; Guanine-G, yellow; Adenine-A, red.

In Figure 3, the electropherogram illustrates the methylation status of the *BRCA1* gene promoter region in ovarian tumor NCI/ADR-RES cells treated for 48 h with araçá-boi extract or gallic acid. The results showed that, regardless of the treatment, the CpG islands analyzed remained methylated, indicating no demethylation in the evaluated region.

The *BRCA1* gene, known for its critical role in DNA damage repair and tumor suppression, is frequently found methylated in ovarian tumor cells. This anomalous methylation in its promoter region leads to the transcriptional silencing of the gene, not only contributing to pathogenesis, but also inducing drug resistance and influencing the prognosis of ovarian cancer [6]. Phytochemicals such as phenolic compounds have been associated with reversing abnormal methylation status in tumor suppressor genes, including *BRCA1*, restoring their expression and promoting cytotoxic effects against tumor cells. This action typically occurs through the inhibition of epigenetic enzymes, such as DNMTs, responsible for the addition and maintenance of methyl groups at CpG dinucleotides, and HDACs, which regulate chromatin compaction levels, directly influencing gene transcription [59,60]. There is growing interest in the use of plant extracts to modulate the DNA methylation of genes involved in critical cellular processes. For instance, the leaf extract of *Vitis vinifera* L. has been shown to significantly alter methylation patterns in the promoters of the *SIRT1* and *HSP47* genes in human fibroblasts exposed to UV radiation, leading to increased expression of these genes, associated with cellular protection and aging delay [61]. Despite these promising advances, there are currently no studies specifically investigating the effects of plant extracts on the methylation of the *BRCA1* promoter in ovarian cancer, highlighting an important gap for future research in this field.

The absence of demethylation in the evaluated promoter region of the *BRCA1* gene can be attributed to several factors. Firstly, although no changes were detected in the methylation status of the specific CpG islands evaluated in the *BRCA1* promoter region, and no reduction in *DNMT1* expression was observed, an upregulation of the *BRCA1* gene was detected in the treated cells. These findings suggest that the modulation of tumor suppressor genes by araçá-boi extract and gallic acid may involve alternative regulatory mechanisms, such as oxidative stress modulation, the activation of transcription factors, histone modifications, or the regulation of non-coding RNAs, rather than occurring through the direct demethylation or inhibition of DNMT1 activity alone. Another possible explanation is that the concentration and duration of treatment may not have been sufficient to induce significant epigenetic changes. Previous studies suggest that both the dose and the exposure time to phenolic compounds directly influence their effects on DNA methylation [62]. In our study, the concentration and treatment duration were determined based on the results of the cell viability assay and subsequently on gene expression analysis. Although these conditions effectively reduced cell viability, they may not have been sufficiently intense or prolonged to induce significant epigenetic changes, such as the demethylation of the *BRCA1* gene promoter region. Additionally, regional specificity may play a critical role, as different CpG islands within the same promoter region can exhibit distinct responses to epigenetic stimuli [63]. The promoter region of the *BRCA1* gene is located approximately 1000 base pairs upstream of exon 1 and includes both the core promoter and regulatory regions essential for the transcriptional regulation of *BRCA1*. The core promoter spans 326 base pairs and contains 25 CpG islands; however, this study focuses on 6 CpG islands over 50 base pairs, positioned from 103 to 153 within the core promoter. Specific CpG regions in tumor suppressor gene promoters are often preferentially methylated in cancer, regardless of treatment [64]. This result may indicate that the CpG islands evaluated in the promoter region of the *BRCA1* gene in NCI/ADR-RES ovarian tumor cells may exhibit an epigenetic stability that resists demethylating stimuli, regardless of the applied treatment. Therefore, exploring other regions within the promoter could provide deeper insights into the broader epigenetic dynamics influencing the methylation status of *BRCA1* in ovarian tumor cells.

## 3. Materials and Methods

### 3.1. Chemicals and Reagents

Folin–Ciocalteu reagent, 2,2-diphenyl-1-picrylhydrazil (DPPH), 2,2′-azinobis-(3-ethylbenzothiazoline-6-sulfonic acid)-diammonium salt (ABTS), TPTZ (2,4,6-tripyridy-s-triazine), 2,2′-azobis(2-methylamidino-propane)-dihydrochloride (AAPH), fluorescein, ethanol, methanol, phenolic compound standards (gallic acid and catechin, grade HPLC, with a purity of ≥96%), (±)-6-hydroxy-2,5,7,8-tetramethylchromane-2-carboxylic acid (Trolox), β-nicotinamide adenine dinucleotide (NADH), phenazine methosulfate (PMS), nitrotetrazolium blue chloride (NBT), sodium hypochlorite solution (NaOCl), and rhodamine 123 were purchased from Sigma-Aldrich (St. Louis, MO, USA). All chemicals used for cell culture, gene expression, and DNA analysis were obtained from Thermo Fisher Scientific (Grand Island, NY, USA) and Invitrogen Life Technologies (Carlsbad, CA, USA). The primers used for gene expression and DNA methylation analyses were synthesized by Exxtend Biotecnologia (Paulínia, SP, Brazil). The other solvents and reagents used in this study were of analytical grade. All solutions were prepared with ultrapure water (18 MΩ cm^−1^) obtained from a Milli-Q water purification system.

### 3.2. Plant Material, Sample Preparation, and Ultrasound-Assisted Extraction

Ripe araçá-boi (*Eugenia stipitata*) fruits were collected during summer at “Kamui Farm” in Ituberá city, in the state of Bahia (Brazil), at geographic coordinates 13°43′56″ S, 39°8′57″ W. The botanical identification and the exsiccate (access number 55,875) were deposited at the Herbarium-UEC of the Agronomic Institute of Campinas, State of São Paulo, Brazil [65]. After sanitation with distilled water, the edible part of the fruit (peel and pulp) was manually separated from the seeds, processed in a home juicer (Philips Walita, Vito RI 6728, Barueri, SP, Brazil), and immediately frozen (−80 °C) for later freeze-drying. The sample was freeze-dried for approximately 52 h (Lyophilizer Series LS E, Terroni Scientific Equipment, São Carlos, SP, Brazil). The freeze-dried product was placed back into plastic bags and vacuum-sealed in a freezer (−18 °C) until analysis. The powders obtained were granulometrically standardized using an electromagnetic sieve shaker 24-mesh (Bertel, model AGMAGB, Caieiras, SP, Brazil). The obtained powders were packed in dark plastic packaging, sealed, and stored at −20 °C.

The phenolic compounds from the edible fraction of araçá-boi were extracted using the method described by Borsoi et al. [16]. Freeze-dried araçá-boi (1 g) was extracted with 15 mL of an ethanol–water mixture (80:20, *v*/*v*). This mixture was subjected to ultrasonic treatment using a UNIQUE UCS-2850 model (25 kHz, 120 W, São Paulo, SP, Brazil) for 10 min at room temperature. Following ultrasonic extraction, the solution was centrifuged at 4000× *g* for 5 min at 5 °C using a Hettich Zentrifugen Rotanta 460R centrifuge (Tuttlingen, Germany). The supernatants were collected after centrifugation, and the residues were re-extracted twice under the same conditions. The combined supernatants were then evaporated under vacuum at 40 °C, and the aqueous phase was concentrated to 50 mL. The resulting araçá-boi extract showed an approximate yield of 3.22% (*w*/*w*) and was stored at −20 °C.

### 3.3. Determination of Total Phenolic Content (TPC) and Total Flavonoid Content (TFC)

The total phenolic content was determined according to the method proposed by Roesler et al. [66]. Briefly, 30 µL of the diluted extract was mixed with 150 µL of 10-fold diluted Folin–Ciocalteau reagent and 120 µL of 7.5% sodium carbonate solution. After 6 min at 45 °C, absorbance was measured at 760 nm on a microplate reader (SpectrostarNano, BMG Labtech, Ortenberg, Germany). A gallic acid standard was used for the analytical curve, and the results were expressed as mg gallic acid equivalent (GAE)/g dw (dry weight).

Total flavonoid content was determined according to the method proposed by Zhishen et al. [67] with modifications. Briefly, 30 μL of the diluted extract was mixed with 110 μL of ultrapure water and 8 μL of 5% sodium nitrite solution. After 5 min of incubation at room temperature, 8 μL of 10% aluminum chloride solution was added and incubated for 6 min at room temperature. Finally, 50 μL of 1 mol/L sodium hydroxide and 70 μL of ultrapure water were added, and the absorbance at 510 nm was measured on a microplate reader (SpectrostarNano, BMG Labtech, Ortenberg, Germany). A catechin standard was used for the analytical curve, and the total flavonoid content was expressed as mg catechin equivalent (CE)/g dw.

### 3.4. Phytochemical Profile by UHPLC-Q-Orbitrap-MS/MS

A Thermo Ultimate 3000 system (Waltham, MA, USA), coupled with a Q-Exactive mass spectrometer equipped with an electrospray ionization (ESI) source, was employed to analyze the phytochemical profile present in the araçá-boi extract following the methods described by Bocker and Silva [68] and Arruda et al. [69]. The mass spectrometer was set to operate in negative electrospray ionization (ESI-) mode, desolvation gas flow at 51 L/min, auxiliary gas flow at 13 L/min, sweep gas flow at 3 L/min, spray voltage at 2.5 kV, capillary temperature at 266 °C, RF lens S at 50, and auxiliary gas temperature at 431 °C. The instrument was used to scan across a mass range of 100–1500 Da at a resolution of 70,000, with an AGC target of 3 × 10^6^ and a maximum injection time (IT) of 100 ms. In MS/MS experiments, a resolution of 17,500 was used, with an AGC target of 1 × 10^5^ and a maximum IT of 50 ms. The top 5 most intense precursor ions were selected for fragmentation using stepped normalized collision energies (NCEs) of 25, 30, and 35 eV, with an isolation window of 3.0 *m*/*z*. The chromatographic separation of the sample was achieved on a Poroshell 120 SB-Aq column (100 × 2.1 mm i.d., 2.7 μm particle size, Agilent Technologies, Santa Clara, CA, USA) with a gradient program at a flow rate of 0.45 mL/min. The column temperature was maintained at 40 °C. The mobile phase consisted of two eluents: A (0.1% formic acid in water) and B (acetonitrile containing 0.1% formic acid). The gradient was performed as follows: 0–1 min, 5% B; 1–10 min, 5–18% B; 10–13 min, 18–70% B; 13–15 min, 70–100% B; 15–17 min, 100% B; 17–19 min, 100–5% B; and 19–22 min, 5% B. Data acquisition and qualitative analysis were performed using Xcalibur 4.3 software. Fragmentation patterns of the detected components were compared against compound databases for plant materials to establish their identities.

### 3.5. Antioxidant Capacity Against Synthetic Free Radicals and Reactive Oxygen Species (ROS)

#### 3.5.1. Scavenging of Synthetic Free Radicals DPPH^•^, ABTS^•+^, and Ferric-Reducing Antioxidant Power (FRAP)

DPPH^•^ scavenging assay was performed according to the method proposed by Roesler et al. [66] with some modifications. Briefly, 50 μL of diluted extract was mixed with 250 μL of 0.004% (*w*/*v*) DPPH in ethanol. The reaction mixture was kept at room temperature for 30 min, and the absorbance at 517 nm was measured on a microplate reader (SpectrostarNano, BMG Labtech, Ortenberg, Germany). A Trolox standard was used for the analytical curve, and the results were expressed as µmol Trolox equivalent (TE)/g dw.

ABTS^•+^ scavenging assay was determined based on the method described by Leite et al. [70]. Firstly, a radical cation ABTS^•+^ solution (7 mmol/L ABTS and 145 mmol/L potassium persulfate) was prepared and incubated in the dark at room temperature overnight. The ABTS^•+^ working solution was diluted with ultrapure water to achieve an absorbance of 0.70 ± 0.02 at 734 nm. The absorbance was measured after the reaction mixture containing 50 µL of diluted extract and 250 µL of ABTS^•+^ solution at 734 nm on a microplate reader (SpectrostarNano, BMG Labtech, Ortenberg, Germany). A Trolox standard was used for the analytical curve, and the results were expressed as µmol Trolox equivalent (TE)/g dw.

The FRAP assay was performed based on the method described by Borsoi et al. [71]. The FRAP solution was prepared by adding 20 mL acetate buffer (0.3 mol/L) at pH 3.6, 2 mL TPTZ solution (10 mmol/L) in 40 mmol/L HCl, and 2 mL ferric chloride solution (20 mmol/L) (10:1:1). The diluted extract (20 µL), FRAP solution (180 µL), and deionized water (60 µL) were mixed and incubated at 37 °C for 30 min before measuring the absorbance at 595 nm using a microplate reader (SpectrostarNano, BMG Labtech, Ortenberg, Germany). A Trolox standard was used for the analytical curve, and the results were expressed as µmol Trolox equivalent (TE)/g dw.

#### 3.5.2. Reactive Oxygen Species (ROS): Peroxyl Radicals (ROO^•^), Hydroxyl Radical (OH^•^), Superoxide Anion (O_2_^•−^), and Hypochlorous Acid (HOCl) Scavenging Assays

The peroxyl radical (ROO^•^) scavenging activity was performed according to the method described by Saliba et al. [72]. The reaction was performed in phosphate buffer (75 mmol/L, pH 7.4) in a 96-well dark microplate. Briefly, 20 µL of diluted extract, 60 µL of the fluorescein solution (508.25 nmol/L), and 110 µL of the AAPH solution (76 mmol/L) were mixed and incubated at 37 °C. Fluorescence was measured with excitation at 485 nm and emission at 528 nm every 10 min for 120 min on a microplate reader (Molecular Devices, Sunnyvale, CA, USA). A Trolox standard was used for the analytical curve, and the results were expressed as µmol Trolox equivalent (TE)/g dw.

The hydroxyl radical (OH^•^) scavenging activity was performed according to the method described by Andrade et al. [73]. The reaction system was formed by the addition of 50 µL of extract (different concentrations), 50 µL of carbonate buffer (0.5 mol/L, pH 10), 50 µL of luminol solution (100 µmol/L) prepared in the carbonate buffer (0.5 mol/L, pH 10), 50 µL of FeCl_2_-EDTA solution (125 and 500 µmol/L), and 50 µL of H_2_O_2_ solution (17.5 mmol/L). The luminescence was measured at 37 °C using a microplate reader (Molecular Devices, LLC, Sunnyvale, CA, USA) after 5 min of incubation. The results were expressed as IC_50_ (μg/mL dw).

The superoxide radical (O_2_^•−^) scavenging activity was performed according to the method described by Saliba et al. [72]. Briefly, each well of the microplate was supplemented with 100 μL of NADH (166 µmol/L), 50 μL of nitroblue tetrazolium (NBT, 107.5 µmol/L), 50 μL of phenazine methosulfate (PMS, 2.7 µmol/L), and 100 μL of different concentrations of the extract dissolved in a 19 mmol/L potassium phosphate buffer solution (pH 7.4) to reach a final volume of 300 µL, and incubated for 5 min. The assay was performed at 25 °C and the fluorescence was measured at 560 nm using a microplate reader (Molecular Devices, LLC, Sunnyvale, CA, USA). The results were expressed as IC_50_ (µg/mL dw).

The hypochlorous acid (HOCl) scavenging activity was performed according to the method described by Saliba et al. [72]. HOCl was prepared using a sodium hypochlorite solution (1% NaOCl), with pH adjusted to 6.2 by adding sulfuric acid solution (10% H_2_SO_4_). The concentration of this solution, diluted in 100 mmol/L phosphate buffer (pH 7.4), was measured at 235 nm using the molar absorption coefficient 100/M/cm for calculation, aiming at a 5 µmol/L HOCl solution. The dihydrorhodamine 123 (DHR) probe was diluted immediately before use at a concentration of 1.25 µmol/L with a 100 mmol/L phosphate buffer at pH 7.4. For the reaction, 100 µL of different extract concentrations, 100 µL phosphate buffer at pH 7.4, 50 µL of the DHR probe, and 50 µL of 5 µmol/L HOCl were added in each microplate well. The assay was performed at 37 °C, and the fluorescence was measured immediately at 528 nm (emission) and 485 nm (excitation) using a microplate reader (Molecular Devices, LLC, Sunnyvale, CA, USA). The results were expressed as IC_50_ (µg/mL dw).

### 3.6. Cell Lines and Culture Conditions

The Chinese hamster ovary (CHO-K1) cell line and human ovarian cancer (NCI/ADR-RES) cell line were kindly provided by Prof. Dr. Ana Lúcia Tasca Gois Ruiz (University of Campinas, UNICAMP). The human ovarian cancer cells (NIH: OVCAR-3) were purchased from the cell bank of Rio de Janeiro (BCRJ, Rio de Janeiro, RJ, Brazil). The cells were grown in flasks containing Roswell Park Memorial Institute (RPMI) (Gibco^TM^) 1640 medium with penicillin/streptomycin (1%) supplemented with 10% fetal bovine serum (RPMI/FBS 10%). The cultures were maintained in 5% carbon dioxide (CO_2_) in a humidified incubator at 37 °C (Revco Habitat, Asheville, NC, USA).

### 3.7. Cell Viability by MTT Assay

To assess the cell viability, we employed a colorimetric assay that has as its principle the reduction of 3-(4,5-dimethyl-2-thiazolyl)-2,5-diphenyl-2H-tetrazolium bromide (MTT) salt, according to Mosmann [74], with some modifications. CHO-K1, NCI/ADR-RES, and OVCAR-3 cells were seeded at a density of 5 × 10^3^ cells per well in a 96-well plate containing 100 μL of full medium and allowed to adhere to the plate for 24 h. After that, the cells were exposed to a different concentration of araçá-boi extract (0.15, 1.5, 15, and 150 μg/mL) and gallic acid (6, 12, 24, and 48 μg/mL). The control group cells received only the culture medium as treatment. All cells were exposed to the treatments for 24, 48, and 72 h. After treatment with araçá-boi extract or gallic acid, the culture medium was aspirated, 100 µL of MTT solution (Sigma-Aldrich, St. Louis, MO, USA) (5 mg/mL in phosphate buffer solution—PBS) was added to each well, and the plates were incubated at 37 °C for 2 h. Then, 85 μL of the medium was removed, 50 μL of DMSO was added to the wells, and this was incubated at 37 °C for 10 min to dissolve the formazan crystals produced by viable cells. Finally, the absorbance was measured at 560 nm using a microplate reader (SpectraMax i3x Multi-Mode, Molecular Devices, Sunnyvale, CA, USA). The results were expressed as a percentage (%) of cell viability compared to the control.

### 3.8. Gene Expression Analysis

#### 3.8.1. Total RNA Isolation

For total RNA extraction, 2 × 10^5^ NCI/ADR-RES cells were seeded in a 60 mm cell culture plastic dish with 4 mL of RPMI-1640 medium (Gibco™) with penicillin/streptomycin (1%) supplemented with 10% of fetal bovine serum (RPMI/FBS 10%). The cultures were maintained in 5% carbon dioxide (CO_2_) in a humidified incubator at 37 °C (Revco Habitat, Asheville, NC, USA). After 24 h, the cells were exposed to different concentrations of araçá-boi extract (1.5 and 15 μg/mL) and gallic acid (24 μg/mL). The control group cells received only the culture medium as treatment. The cells were collected in TRIzol (500 µL) after 48 h. The RNA extraction protocol was performed according to Chomczynski and Sacchi [75]. Briefly, 80 µL of chloroform was added to the TRIzol lysate and mixed thoroughly by vortexing. After 3 min, the samples were centrifuged at 10,000× *g* for 15 min at 4 °C (Centrifuge 5427 R, Eppendorf, Hamburg, Germany) and the upper aqueous phase containing RNA was collected in a new tube. Then, 200 µL of isopropanol was added to the sample, mixed, and kept at room temperature for 10 min before centrifugation at 10,500× *g* for 10 min at 4 °C. The supernatant was discarded, and the pellet was washed with 200 µL ethanol solution (75%) and then centrifuged at 5200× *g* for 5 min at 4 °C. The pellet was air-dried and 30 µL RNase-free water was added. Finally, the RNA was quantified with a NanoDrop^®^ 2000 spectrophotometer (Thermo Fisher Scientific, Waltham, MA, USA).

#### 3.8.2. cDNA Synthesis and Primer Design

The cDNA synthesis was performed according to Invitrogen’s M-MLV Reverse Transcriptase (200 U/μL) instructions with 700 ng of total RNA per reaction. The sequences of the internal control gene, *GAPDH*, as well as the genes of interest—*BRCA1*, *RASSF1A*, *CDKN2A*, *DNMT1*, and *HDAC1*—were designed using the UCSC Genome Browser ((https://genome.ucsc.edu) accessed on 15 February 2023) and Primer3Plus Browser ((https://www.primer3plus.com) accessed on 15 February 2023). To ensure the quality and compatibility of the primers, the ‘NetPrimers’ software (Premier Biosoft, Palo Alto, CA, USA) and OligoAnalyzer™ Tool ((https://www.idtdna.com/pages/tools/oligoanalyzer) accessed on 15 February 2023) were employed. Table 3 presents a list of the primer names, their sequences, sizes, amplicon length (in bp), and corresponding annealing temperatures.

#### 3.8.3. Quantitative Reverse Transcription Polymerase Chain Reaction (qRT-PCR)

The RT-qPCR was performed using a FastStart Universal SYBR Green Master Mix (Roche Diagnostic GmbH, Mannheim, Germany), according to the instructions provided by the manufacturer. Briefly, 5 μL of cDNA, 10 μL of FastStart Universal SYBR Green Master (ROX), and 5 µL of forward and reverse primers (200 nM) were mixed. Then, the reactions were performed using universal cycling conditions in the StepOnePlus™ real-time PCR system (Applied BioSystems, Foster City, CA, USA), and the parameters were as follows: 2 min at 50 °C (UDG pretreatment) and 10 min at 95 °C, followed by 40 rounds of 15 s at 95 °C and 60 s at 60 °C. A melting curve (15 s at 95 °C followed by 60–95 °C at increments of 1.0 °C) was generated to verify the specificity of the primer amplification. The relative expression of each gene was represented as the fold expression in relation to the control and calculated using the comparative 2^(−ΔΔCT)^ value. GAPDH was used as the housekeeping gene to normalize gene expression.

### 3.9. DNA Methylation Analysis

#### 3.9.1. Bisulfite Conversion of DNA

For the bisulfite conversion of DNA, 2 × 10^5^ NCI/ADR-RES cells were seeded into 6-well plates with 2 mL of RPMI-1640 medium (Gibco™) with penicillin/streptomycin (1%) supplemented with 10% of fetal bovine serum (RPMI/FBS 10%). The cultures were maintained in 5% carbon dioxide (CO_2_) in a humidified incubator at 37 °C (Revco Habitat, Asheville, NC, USA) for 24 h to adhere to the plate. Afterward, the cells were exposed to araçá-boi extract (15 μg/mL) and gallic acid (24 μg/mL) for 48 h. The control group cells received only the culture medium as treatment. The EZ DNA Methylation-Direct™ Kit (Zymo Research Corporation, Irvine, CA, USA) was used for DNA extraction and direct bisulfite conversion, according to the instructions provided by the manufacturer. The recovered bisulfite-treated DNA was quantified with a NanoDrop^®^ 2000 spectrophotometer (Thermo Fisher Scientific, Waltham, MA, USA).

#### 3.9.2. Primer Design and PCR-Amplification of the Bisulfite-Treated DNA

The promoter region of the *BRCA1* gene was obtained from the UCSC Genome Browser ((https://genome.ucsc.edu) accessed on 15 February 2023) and the primers were obtained using the bisulfite primer design tool (Bisulfite Primer Seeker, Zymo Research Corporation, Irvine, CA, USA). Furthermore, to check the quality and compatibility of the primers, ‘NetPrimers’ software (Premier Biosoft, Palo Alto, CA, USA) and OligoAnalyzer™ Tool ((https://www.idtdna.com/pages/tools/oligoanalyzer) accessed on 15 February 2023) were used. The *BRCA1* forward primer was 5′-TTTAGTTTTAGGAGTTTGGGGTAAGTAG-3′ and reverse 5′-CCTTAAACTTCTCCAAACCCTCTTAATA-3′. PCR was performed on a Mastercycler ep (Eppendorf AG, 22331 Hamburg, Germany) for bisulfite-converted DNA and was conducted in a final volume of 50 μL. The reaction consisted of 5 μL high-fidelity PCR buffer (10×), 0.2 μL of 5 U/rxn Platinum^®^ Taq DNA polymerase high fidelity (Invitrogen™, Carlsbad, CA, USA), 1 μL bisulfite-treated genomic DNA (40 ng/μL), 4.5 μL of each primer (10 μM), 1 μL dNTPs (10 mM), 2 μL MgSO_4_ (2 mM), and autoclaved water to complete the volume, according to the instructions provided by the manufacturer. The PCR conditions for the *BRCA1* gene were 94 °C for 2 min, followed by 40 cycles of 94 °C for 15 s, 54 °C for 30 s, and 72 °C for 1 min. PCR amplicons were examined by gel electrophoresis on 1% agarose for the presence of single bands at the expected size.

#### 3.9.3. Purification and Sanger Sequencing

The previously obtained PCR products were purified to remove unincorporated nucleotides and excess primers, using the Wizard^®^ SV Gel and PCR Clean-Up System (Promega Corporation, Madison, WI, USA), according to the manufacturer’s recommendations. After purification of the PCR products, the samples were quantified (ng/μL) with a NanoDrop^®^ 2000 spectrophotometer (Thermo Fisher Scientific, Waltham, MA, USA). Sequencing reactions were performed using the BigDye Terminator v3.1 Cycle Sequencing Kit (Applied Biosystems™, Waltham, MA, USA), with the same primers used in the PCR, and performed on 3730xl DNA Analyzer (Applied Biosystems™, Waltham, MA, USA). The runs were made in 36 cm capillaries using the POP7 polymer. The sequences were processed and analyzed using Genious™ software version 4.8.5.

### 3.10. Statistical Analysis

The results are expressed as the mean ± standard deviation value of at least three independent experiments using GraphPad Prism software version 9. Statistical analyses for the cellular assay were acquired using two-way ANOVA, followed by Dunnett’s post hoc multiple comparison test. Gene expression data were submitted to two-way ANOVA, followed by the Bonferroni test. Significant differences are symbolized using *p*-values of * *p* < 0.05, ** *p* < 0.01, *** *p* < 0.001, and **** *p* < 0.0001.

## 4. Conclusions

For the first time, we demonstrated the potential of araçá-boi extract in modulating epigenetic pathways involved in the onset and progression of ovarian cancer. Araçá-boi extract exhibited remarkable antioxidant activity, acting as an effective scavenger of free radicals and reactive oxygen species, particularly hydroxyl and peroxyl radicals, suggesting its therapeutic potential, especially in conditions associated with oxidative stress. Cytotoxicity assays revealed that the araçá-boi extract significantly reduced cell viability in ovarian cancer cells (NCI/ADR-RES and OVCAR-3) without inducing cytotoxicity in normal CHO-K1 cells, highlighting its selective anticancer effect. Notably, both the araçá-boi extract and gallic acid were more effective against the NCI/ADR-RES cell line. Gene expression analysis showed that the araçá-boi extract upregulated tumor suppressor genes (*BRCA1* and *RASSF1A*) and the histone deacetylation gene (*HDAC1*), while gallic acid induced the expression of *BRCA1*, *HDAC1*, and *CDKN2A*, suggesting the activation of DNA repair mechanisms and the induction of cell cycle arrest. These biological activities can be attributed, at least in part, to the rich and diverse phytochemical profile of the araçá-boi extract, particularly its high content of phenolic acids and flavonoids. Despite these promising findings, neither the araçá-boi extract nor gallic acid promoted demethylation in the evaluated promoter region of the *BRCA1* gene in NCI/ADR-RES cells, indicating a possible epigenetic stability of this region. Nevertheless, the observed reduction in cell viability and the modulation of key genes suggest that other molecular and epigenetic mechanisms are likely involved. Altogether, these findings reinforce the therapeutic potential of araçá-boi extract and its phenolic compounds as promising candidates for developing complementary strategies in ovarian cancer treatment. Further studies, particularly in vivo investigations and broader epigenetic and mechanistic analyses, are essential to deepen our understanding of the molecular pathways involved and to validate the clinical applicability of this extract and its bioactive compounds.

## Figures and Tables

**Table 1:** Total phenolic content, total flavonoid content, and antioxidant capacity against synthetic free radicals and reactive oxygen species (ROS) in araçá-boi extract.

Analysis	Parameters	Araçá-Boi Extract
Phytochemicals	Total phenolics (mg GAE/g dw)	25.90 ± 1.37
	Total flavonoids (mg CE/g dw)	6.53 ± 0.19
Synthetic free radical	DPPH (μmol TE/g dw)	68.78 ± 7.03
	ABTS (μmol TE/g dw)	155.52 ± 8.08
	FRAP (μmol TE/g dw)	161.77 ± 10.21
Reactive oxygen species (ROS)	ROO^•^ (μmol TE/g dw)	366.13 ± 19.39
	OH^•^ (IC_50_ µg/mL dw)	1.91 ± 0.20
	O_2_^•−^ (IC_50_ µg/mL dw)	3534.33 ± 111.99
	HOCl (IC_50_ µg/mL dw)	307.66 ± 30.08

CE: catechin equivalents; dw: dry weight; FRAP: ferric reducing antioxidant power; GAE: gallic acid equivalents; HOCl: hypochlorous acid scavenging activity; IC_50_: extract concentration that resulted in a 50% reduction in radical concentration compared to the control; O_2_^•−^: superoxide radical scavenging activity; OH^•^: hydroxyl radical scavenging activity; ROO^•^: peroxyl radical scavenging activity; TE: Trolox equivalents.

**Table 2:** Phytochemicals identified or tentatively annotated in araçá-boi extract using UHPLC-Q-Orbitrap-MS/MS in negative ion mode.

N. °	R.T. (min)	Identified/Tentatively Annotated Compound	Molecular Formula	Observed *m/z* Value	Theoretical *m/z* Value	Error (ppm)	Characteristic MS/MS Fragments
	*Organic acid and derivatives*						
1	0.68	Quinic acid	C_6_H_12_O_6_	191.0566	191.0556	5.23	191.0568, 173.0456, 127.0408, 93.0347, 85.0294
2	0.74	Malic acid	C_4_H_6_O_5_	133.0144	133.0137	5.26	115.0038, 71.0135
3	0.83	Citric acid	C_6_H_8_O_7_	191.0201	191.0192	4.71	129.0192, 111.0090
4	0.90	Shikimic acid	C_7_H_10_O_5_	173.0452	173.0450	1.16	155.0004, 111.0087, 93.0350
5	0.90	Succinic acid	C_4_H_6_O_4_	117.0180	117.0188	−6.84	99.0087, 73.0295
6	0.93	Hydroxyadipic acid	C_6_H_10_O_5_	161.0457	161.0450	4.35	101.0245, 99.0451
7	1.38	Ascorbic acid	C_6_H_8_O_6_	175.0251	175.0243	4.57	115.0037, 87.0090, 71.0138
8	1.23	Pantothenic acid (vitamin B5)	C_9_H_17_NO_5_	218.1033	218.1028	2.29	146.0829, 88.0402
9	4.43	Tuberonic acid hexoside	C_18_H_28_O_9_	387.1662	387.1655	1.81	207.1024, 163.1134
10	10.87	12-hydroxyjasmonoyl-isoleucine	C_18_H_29_NO_5_	338.1984	338.1967	5.03	130.0876
	*Phenolic acids and derivatives*						
11	0.99	Gallic acid glucoside	C_13_H_16_O_10_	331.0678	331.0665	3.93	271.0489, 211.0272, 169.0155
12	1.14	Gallic acid	C_7_H_6_O_5_	169.0146	169.0137	5.33	169.0146, 125.0245, 107.0137, 97.0299, 79.0188
13	1.39	Salicylic acid isomer 1	C_7_H_6_O_3_	137.0244	137.0239	3.65	93.0349
14	1.62	Hydroxybenzoic acid hexoside	C_13_H_16_O_8_	299.0775	299.0767	2.67	137.0252
15	1.70	Peduncalagin isomer 1	C_34_H_24_O_22_	783.0698	783.0681	2.17	300.9982, 275.0201
16	1.99	Vanillic acid hexoside isomer 1	C_14_H_18_O_9_	329.0886	329.0872	4.25	329.0946, 167.0350, 152.0115, 123.0455, 108.0220
17	2.06	Protocatechuic acid xyloside	C_12_H_14_O_8_	285.0620	285.0610	3.51	153.0192, 152.0112, 123.4724, 109.0290, 108.0222
18	2.12	Vanillic acid hexoside isomer 2	C_14_H_18_O_9_	329.0887	329.0872	4.56	329.0908, 167.0358, 123.0451
19	2.19	Salicylic acid isomer 2	C_7_H_6_O_3_	137.0243	137.0239	2.92	93.0341
20	2.26	Galloyl shikimic acid	C_14_H_14_O_9_	325.0573	325.0560	4.00	169. 0154, 125.0252
21	2.33	Caffeic acid hexoside isomer 1	C_15_H_18_O_9_	341.0885	341.0873	3.52	179.0360, 161.0255, 135.0455
22	2.59	Peduncalagin isomer 2	C_34_H_24_O_22_	783.0710	783.0681	3.70	300.9995, 275.0200
23	2.62	Coumaric acid isomer 1	C_9_H_8_O_3_	163.0403	163.0395	4.91	162.8391, 119.0504
24	2.62	p-coumaric acid hexoside isomer 1	C_15_H_18_O_8_	325.0933	325.0923	3.08	163.0403, 119.0504
25	2.74	Syringic acid hexoside isomer 1	C_15_H_20_O_10_	359.0995	359.0978	4.73	197.0839, 138.3060, 123.0091
26	2.78	Caffeic acid hexoside isomer 2	C_15_H_18_O_9_	341.0886	341.0873	3.81	179.0361, 135.0459
27	2.95	Vanillic acid hexoside isomer 3	C_14_H_18_O_9_	329.0887	329.0872	4.56	167.0352, 123.0456
28	3.10	Coumaric acid isomer 2	C_9_H_8_O_3_	163.0403	163.0395	4.91	162.8406, 119.0507
29	3.12	p-coumaric acid hexoside isomer 2	C_15_H_18_O_8_	325.0939	325.0923	4.92	163.0410, 145.0301, 119.0498
30	3.22	Caffeic acid hexoside isomer 3	C_15_H_18_O_9_	341.0883	341.0873	2.93	179.0356, 135.0453
31	3.28	Coumaric acid isomer 3	C_9_H_8_O_3_	163.0402	163.0395	4.29	162.8410, 119.0506
32	3.31	p-coumaric acid hexoside isomer 3	C_15_H_18_O_8_	325.0938	325.0923	4.61	163.0408, 145.0292, 119.0506
33	3.72	Vanillic acid hexoside isomer 4	C_14_H_18_O_9_	329.0887	329.0872	4.56	167.0357, 123.0456, 108.0218
34	3.81	Digalloyl hexoside isomer 1	C_20_H_20_O_14_	483.0797	483.0775	4.55	313.0592, 271.0473, 211.0240, 169.0148
35	4.10	Digalloyl hexoside isomer 2	C_20_H_20_O_14_	483.0796	483.0775	4.35	169.0144, 125.0241
36	4.14	p-coumaric acid hexoside isomer 4	C_15_H_18_O_8_	325.0939	325.0923	4.92	163.0392, 119.0498
37	4.15	Ferulic acid hexoside	C_16_H_20_O_9_	355.1041	355.1029	3.38	193.0498, 175.0413, 134.0370
38	4.15	Di-O-galloyl-rhamnose	C_20_H_20_O_13_	467.0806	467.0826	−4.28	315.0174, 169.0143, 125.0251
39	4.16	Ferulic acid	C_10_H_10_O_4_	193.0508	193.0501	3.63	134.0376
40	5.11	Coumaric acid isomer 4	C_9_H_8_O_3_	163.0402	163.0395	4.29	162.8396, 119.0506
41	5.17	Trans-cinnamic acid	C_9_H_8_O_2_	147.0453	147.0446	4.76	147.0457, 103.0549
42	5.88	Syringic acid hexoside isomer 1	C_15_H_20_O_10_	359.0964	359.0978	−3.90	197.0831, 153.0923
43	6.10	Caffeoylshikimic acid	C_16_H_16_O_8_	335.0784	335.0770	4.18	179.0359, 161.0258, 135.0447
44	6.34	Tri-O-galloyl-glucose	C_27_H_24_O_18_	635.0926	635.0884	6.61	465.0686, 313.0588, 169.0142, 125.0249
45	7.77	Mirciaphenone B	C_21_H_22_O_13_	481.0994	481.0982	2.49	313.0557, 169.0147
46	9.25	Cis-Cinnamic acid	C_9_H_8_O_2_	147.0452	147.0446	4.08	147.0459, 103.0549
	*Flavonoids and derivatives*						
47	3.24	Taxifolin isomer 1	C_15_H_12_O_7_	303.0514	303.0505	2.97	285.0428, 217.0512, 175.0395, 125.0245
48	3.44	Taxifolin isomer 2	C_15_H_12_O_7_	303.0513	303.0505	2.64	285.0403, 217.0499, 175.0410, 125.0243
49	4.74	(Epi)catechin	C_15_H_14_O_6_	289.0723	289.0712	3.81	245.0483, 221.0465, 151.0033, 137.0254, 125.0251
50	5.28	Dihydroquercetin hexoside	C_21_H_22_O_12_	465.1045	465.1068	−4.95	285.0390, 151.0038
51	6.99	Taxifolin isomer 3	C_15_H_12_O_7_	303.0515	303.0505	3.30	285.0417, 175.0397, 125.0250
52	7.52	Myricetin-3-O-galactoside	C_21_H_20_O_13_	479.0848	479.0826	4.59	317.0311, 316.0230
53	8.33	Quercetin-3-O-galloyl hexoside isomer 1	C_28_H_24_O_16_	615.1002	615.0986	2.60	463.0884, 301.0350, 300.0306, 169.0145, 151.0046
54	8.68	Quercetin-3-O-galloyl hexoside isomer 2	C_28_H_24_O_16_	615.1002	615.0986	2.60	463.0849, 301.0344, 300.0313, 169.0144, 125.2322
55	8.74	Myricetin-3-O-rhamnoside (myricetrin)	C_21_H_20_O_12_	463.0895	463.0877	3.89	316.0224, 137.0305
56	8.80	Quercetin maloyl hexoside	C_25_H_24_O_16_	579.1018	579.0986	5.53	301.0336, 300.0305
57	8.98	Quercetin-3-O-galactoside	C_21_H_20_O_12_	463.0899	463.0877	4.75	301.0342, 300.0311, 179.1588, 151.0037
58	9.08	Quercetin-3-O-glucuronide	C_21_H_18_O_13_	477.0690	477.0669	4.40	302.0402, 301.0370, 178.9986, 151.0045
59	9.23	Phloretin-C-diglycoside	C_27_H_34_O_15_	597.1830	597.1820	1.67	387.1130, 357.1005, 345.0978, 315.0868, 209.0453
60	9.24	Quercetin-3-O-glucoside	C_21_H_20_O_12_	463.0900	463.0877	4.97	301.0358, 300.0278, 178.9999, 151.0037
61	9.46	Naringenin	C_15_H_12_O_5_	271.0618	271.0607	4.06	177.0196, 151.0033, 119.0509, 107.0135
62	10.08	Quercetin-3-O-arabinoside	C_20_H_18_O_11_	433.0795	433.0771	5.54	301.0359, 300.0279, 271.0620, 151.0031
63	10.16	Kaempferol-3-O-galactoside (trifolin)	C_21_H_20_O_11_	447.0938	447.0927	2.46	285.0390, 284.0330, 255.0307
64	10.18	Phlorizin	C_21_H_24_O_10_	435.1302	435.1291	2.53	273.0791, 167.0364
65	10.49	Kaempferol 7-(6′-galloyl glucoside)	C_28_H_24_O_15_	599.1061	599.1037	4.01	285.0420, 284.0360, 169.0143
66	10.69	Kaempferol-3-O-glucoside (astragalin)	C_21_H_20_O_11_	447.0948	447.0927	4.70	285.0410, 284.0350, 255.0322
67	10.79	Quercetin-3-O-rhamnoside (quercetrin)	C_21_H_20_O_11_	447.0948	447.0927	4.70	301.0341, 300.0298, 271.0249, 255.0325, 151.0048
68	11.85	Kaempferol-3-O-rhamnoside (afzelin)	C_21_H_20_O_10_	431.0994	431.0978	3.71	286.0453, 285.0408, 284.0348, 255.0320, 227.0348
69	12.11	Quercetin deoxyhesoxylhexoside	C_27_H_30_O_16_	609.1442	609.1456	−2.30	301.0333, 300.0320
70	12.18	Quercetin-3-O-acetyl rhamnoside	C_23_H_22_O_12_	489.1056	489.1033	4.70	301.0337, 300.0306, 271.0245, 255.0322
71	12.18	Quercetin 3-O-hexuronide-7-O-hexoside	C_27_H_28_O_18_	639.1212	639.1197	2.35	301.0344, 300.0289, 151.0700
72	12.35	Quercetin	C_15_H_10_O_7_	301.0359	301.0348	3.65	301.0376, 179.0004, 151.0043, 121.0305, 107.0144
73	12.49	Quercetin-3,7-O-dirhamnoside	C_27_H_30_O_15_	593.1483	593.1506	−3.88	301.0356, 300.0275, 271.0263, 255.0301, 151.0035

**Table 3:** Sequences of primers used for quantitative reverse transcription polymerase chain reaction (qRT-PCR).

Gene Names	Forward (5′→3′)	Reverse (5′→3′)	Amplicon (bp)
*BRCA1*	CTGGACAGAGGACAATGGCT	GTGGGGGATCTGGGGTATCA	139
*RASSF1A*	ACCCCTCTGCCCTCATTACT	TTCTGTCTGCACCACTCCTG	89
*DNMT1*	TTCAGCACAACCGTCACCAA	GTCCAGGATGTTGCCGAAGA	147
*HDAC1*	TTCTTCCCCAACCCCTCAGA	GGCCTTGGTTTCTGTCCCTG	99
*CDKN2A*	TAAGGGGAATAGGGGAGCGG	ACTGCGAGAACCACATGTCT	149
*GAPDH*	ACCCACTCCTCCACCTTTGA	CTGTTGCTGTAGCCAAATTCGT	101

bp: base pairs.

## Data Availability

Data are contained within the article.
